# Exploration of the mechanism underlying the therapeutic effect of electroacupuncture at chengshan acupoint on post-hemorrhoidectomy anal pain: Insights from the mAChRs/IP3-Ca^2+^-CaM signaling pathway

**DOI:** 10.1016/j.clinsp.2024.100485

**Published:** 2024-09-23

**Authors:** Yang Song, Yang Wang, Ming Li, Yujuan Wang, Tianshu Xu

**Affiliations:** a Department of Traditional Chinese Medicine, Nanjing Drum Tower Hospital Clinical College of Nanjing University of Chinese Medicine, Nanjing, Jiangsu, China; b Department of Traditional Chinese Medicine, Nanjing Drum Tower Hospital, Nanjing, Jiangsu, China

**Keywords:** Anal pain, Muscarinic acetylcholine receptors, IP3-Ca^2+^-CaM signaling pathway, Electroacupuncture, Postoperative pain management

## Abstract

**Objective:**

In the context of postoperative anal pain, understanding the intricate mechanisms and effective interventions is paramount. This study investigates the role of Muscarinic Acetylcholine Receptors (mAChRs) and the IP3-Ca^2+^-CaM signaling pathway in a rat model of postoperative anal pain, exploring the potential analgesic effects of electroacupuncture.

**Methods:**

Comprehensive approaches involving mechanical sensitivity assays, Western blotting, immunohistochemistry, and intracellular calcium concentration measurement were used.

**Results:**

The authors found elevated mAChRs expression in the postoperative pain model. Antagonizing mAChRs reduced pain sensitivity and attenuated the IP3-Ca^2+^-CaM pathway. Remarkably, electroacupuncture treatment further mitigated pain, potentially by suppressing this signaling cascade.

**Interpretation:**

These findings reveal a novel connection between mAChRs and the IP3-Ca^2+^-CaM pathway in postoperative anal pain and suggest electroacupuncture as a promising avenue for pain relief through these mechanisms, offering insights into innovative strategies for postoperative pain management.

## Highlights

• Expression of mAChRs was elevated in the rat model of postoperative pain.
• Antagonizing mAChRs reduced pain sensitivity and attenuated the IP3-Ca^2+^-CaM pathway.
• Electroacupuncture further mitigated pain by suppressing IP3-Ca^2+^-CaM signaling.

## Introduction

Hemorrhoidectomy is a surgery used to treat hemorrhoids when symptoms are severe or conservative treatments fail to provide relief.^1^ Pain and infection are the most common risks associated with this type of surgery.^2^ The incidence of moderate to severe pain following conventional hemorrhoidectomy reported in the literature could be as high as 65%.^3^ Pain medications and stool softeners may also be involved in the recovery process.^4^ Pain usually lasts for about 1 week after surgery, but it can vary depending on the type of hemorrhoidectomy performed and other factors.^5^

Among all interventions, electroacupuncture has emerged as a potentially promising avenue.^6^^,^^7^ Rooted in ancient Chinese medicine, electroacupuncture involves the precise insertion of fine needles into specific acupoints, coupled with controlled electrical stimulation.^8^ Its utility in pain mitigation has gained substantial attention due to its potential to modulate neural pathways and trigger the release of endogenous opioids.^9^

The mAChRs/IP3-Ca^2+^-CaM signaling pathway is involved in pain relief. Muscarinic Acetylcholine Receptors (mAChRs) are a type of acetylcholine receptor that form G protein-coupled receptor complexes in the cell membranes of certain neurons and other cells.^10^ When activated, these receptors can trigger the release of intracellular Calcium ions (Ca^2+^) through the Inositol 1,4,5-Trisphosphate Receptor (IP3R), a calcium channel located on the endoplasmic reticulum.^11^ This increase in intracellular calcium can then activate Calmodulin (CaM), a protein that binds to and regulates the activity of various enzymes and other proteins.^12^ This signaling pathway has been shown to play a role in pain relief, although the exact mechanisms are not yet fully understood.

The present study endeavors to illuminate these mechanisms by meticulously exploring the intricate interplay between electroacupuncture, cholinergic signaling, and postoperative anal pain modulation. Furthermore, the authors delve into the potential modulation of intracellular signaling cascades, notably the IP3-Ca^2+^-CaM pathway, to elucidate the mechanistic underpinnings of electroacupuncture's analgesic efficacy.

As the authors embark on this investigative journey, we aim to uncover the nuanced relationships that underscore electroacupuncture's capacity to alleviate postoperative anal pain. By doing so, the authors aspire to bridge the translational gap between ancient wisdom and modern medicine, propelling us toward enhanced patient care and improved surgical outcomes.

## Materials and methods

### Animal model

Two strains, male Sprague-Dawley (SD) rats and mAChRs(-/-) rats, were employed. Ethical approval was obtained from the Animal Ethics Committee. Rats, aged 8‒10 weeks and weighing 250‒300 grams, were sourced from Cyagen (China) and individually housed under standard conditions with ad libitum access to food and water. Rats were anesthetized using isoflurane, and a longitudinal incision of approximately 1 cm in length was made in the perianal region. The wound was sutured using 5‒0 absorbable sutures and a simple interrupted technique. Rats were allowed to recover in individual cages with standard bedding and ad libitum access to rodent chow and water. All rats underwent routine postoperative symptomatic treatment such as infection prevention and hemostasis.

### Electroacupuncture treatment

In the electroacupuncture treatment group, following successful model establishment, rats were securely positioned on a fixation apparatus. Guided by the “Experimental Acupuncture Point Atlas for Laboratory Animals” formulated by the China Association of Acupuncture and Moxibustion, the “Chengshan” acupoint was meticulously selected. A single-use fine needle measuring 0.18 mm in diameter and 13 mm in length was vertically inserted to a depth of 4 mm at the designated acupoint. The needle was then connected to an electronic acupuncture device programmed to deliver continuous-wave stimulation. The stimulation frequency was maintained at approximately 10 Hz, and the intensity was carefully adjusted to 0.5 mA, ensuring a mild muscular twitch without rat discomfort or resistance. Electroacupuncture treatment was administered daily for 30 minutes per session, spanning a total of 7 consecutive days. This therapeutic regimen targeted the “Chengshan” acupoint, aiming to harness the potential analgesic effects of electroacupuncture in mitigating post-anal incision pain. The adherence to standardized procedures and carefully selected parameters underscored the scientific rigor and meticulousness in administering electroacupuncture as a potential intervention.

### Evaluation of mechanical pain sensitivity

A meticulous evaluation of mechanical pain sensitivity was accomplished through the implementation of the von Frey filament method.^13^ This procedure involved assessing two distinct anatomical locations: the immediate incision site and a spatially distant area positioned 15 cm away from the incision site. Each rat was placed on an elevated mesh platform and subjected to a series of calibrated von Frey filaments with varying bending forces. At the incision site and the distant area, the threshold force required to elicit a withdrawal response was recorded. This procedure was conducted at specific time points post-surgery to track the dynamic changes in mechanical pain sensitivity.

### Anal sphincter resting pressure measurements

Employing custom-designed probe holders accommodating sleeve sensors of varying diameters (3 mm, 4.5 mm, 6 mm, and 9 mm), the measurements were tailored to the size of rat fecal pellets, which typically measure 10‒11 mm. Sleeve sensors were positioned within the holders, oriented towards the posterior midline direction of the anal canal. Significantly, these sensors detected the maximal pressure along their length, effectively capturing the overall pressure within the anal canal, regardless of specific muscle involvement. Electrical stimulation was introduced through a pulse generator (S48, Grass Technologies, USA) linked to a constant current unit (CCU1A, Grass Technologies, USA). Currents ranging from 1 to 5 mA were applied at 50 Hz frequency, with a pulse duration of 0.2 ms, enabling the assessment of individual muscle components' contributions to anal canal pressure. Furthermore, the roles of the Internal Anal Sphincter (IAS) and External Anal Sphincter (EAS) were further elucidated by applying sodium nitroprusside (SNP; Sigma Chemical, St. Louis, MO) at 1.5 μg/kg and PB (0.4 mg/kg).

### Quantification of fecal pellet excretion

Following the surgical intervention, individual rat subjects were housed in metabolic cages for a designated postoperative period of 6 hours. Fecal pellets excreted during this specific timeframe were diligently collected and preserved for analysis. The collected fecal pellets were subjected to a meticulous manual counting process, ensuring a precise enumeration of the pellets expelled by each subject within the defined 6-hour postoperative window.

### Acetylcholine (Ach) level measurement

Rats were gently restrained to facilitate blood sample collection. Blood was carefully drawn from the tail vein using a calibrated micro-sampling system. The collected blood samples were subjected to centrifugation to obtain plasma, which was stored at -80°C until analysis. The analysis of Ach levels was conducted using the EnzyChrom™ Acetylcholine Assay Kit (ab65345, Abcam, UK). Plasma samples underwent a precise extraction procedure using the provided extraction buffer, isolating Ach from the biological matrix. The extracted Ach was subjected to enzymatic reactions as outlined in the assay protocol, yielding specific detectable products. Quantification of Ach levels was achieved by comparing the absorbance of the enzymatic reaction products at 570 nm against standard calibration curves generated using known concentrations of Ach standards (included in the kit). The concentrations were normalized to the protein content of the samples using the DC Protein Assay Kit (500-0116, Bio-Rad, USA), yielding the nmol/mg protein unit.

### Western blot (IP3, CAM, M2 mAChR, M3 mAChR, beta-actin)

Protein concentrations were determined using the BCA Protein Assay Kit (23225, Thermo Fisher, USA). Equal amounts of protein were loaded onto SDS-PAGE gels and separated by electrophoresis. Proteins were then transferred onto PVDF membranes using a semi-dry transfer system. The membranes were blocked in 5% non-fat milk solution and subsequently probed with primary antibodies specific to IP3 (ab5804, Abcam, UK), CAM (MA3-917, Thermo Fisher, USA), M2 mAChR (sc-33712, Santa Cruz, USA), M3 mAChR (sc-518107, Santa Cruz, USA), and beta-actin (A5316, Sigma-Aldrich, USA). After incubation with appropriate secondary antibodies, protein bands were visualized using Enhanced Chemiluminescence (ECL) reagents (1705061, Bio-Rad, USA). Bands were captured using a gel documentation system and quantified using suitable software.

### Immunohistochemistry (IHC) analysis

Striatal tissue samples were meticulously collected and fixed in a 4% paraformaldehyde solution. Following fixation, tissues were embedded in paraffin blocks and sectioned into thin slices (5 μm thickness). The tissue sections were incubated with primary antibodies targeting M2 mAChR (ab109226, Abcam, UK) and M3 mAChR (ab87199, Abcam, UK). The specific binding of primary antibodies to target proteins was facilitated through secondary antibodies (ab150077, Abcam, UK). Following the application of HRP substrate or fluorescence-compatible dyes, the stained tissue sections were observed under a light microscope.

### Quantification of intracellular calcium ion concentration

Samples were loaded with the fluorescent calcium indicator Fluo-4 AM (F14201, Thermo Fisher, USA), enabling Ca^2+^ binding and subsequent fluorescence emission. A fluorescence microplate reader (BioTek) equipped with excitation (485 nm) and emission (520 nm) filters were used for measurements. Microplate wells were coated with Corning® Cell-Tak (TM) Cell and Tissue Adhesive (CLS354240, Corning, USA) to facilitate tissue attachment and stability during measurement. Upon excitation, the emitted fluorescence from Fluo-4 AM was quantified. Fluorescence intensity changes directly correlated with intracellular Ca^2+^ concentration fluctuations induced by experimental manipulations.

### Isolation of anal sphincter smooth muscle cells

Rat anal sphincter tissues were harvested with precision and transferred to a physiological salt solution at 4°C. The tissues were cleaned of surrounding connective tissue and blood vessels and subsequently cut into smaller segments. The tissue segments were subjected to enzymatic digestion using collagenase type II (LS004176, Worthington Biochemical Corporation, USA) and papain (P3125, Sigma-Aldrich, USA). This process was carried out at a controlled temperature under gentle agitation. Following enzymatic digestion, tissues were gently triturated to facilitate cell dispersal. The dispersed cell suspension was filtered through a cell strainer to remove undigested tissue fragments. The cell suspension was subjected to centrifugation to obtain a pellet of smooth muscle cells. The supernatant was removed, and the pellet was resuspended in an appropriate culture medium.

### Cell treatment with antagonists

Cultured anal sphincter smooth muscle cells were maintained under controlled conditions. To investigate the role of muscarinic acetylcholine receptors (mAChRs), cells were treated with selective antagonists: atropine (A0132, Sigma-Aldrich,USA) at a concentration of 10 μM for non-specific mAChR blockade, 4-DAMP (0929, Tocris Bioscience, USA) at a concentration of 1 μM for M3 mAChR inhibition, and AF-DX 116 (0674, Tocris Bioscience, USA) at a concentration of 2 μM for M2 mAChR inhibition. All incubation lasted for 1h. Following treatment, cellular responses were evaluated through Western blotting.

### Statistical analysis

Prior to statistical testing, data distributions were assessed for normality using the Shapiro-Wilk test. For comparisons between the two groups, an unpaired Student's *t*-test or Mann-Whitney *U* test was employed, depending on the data distribution. For multiple group comparisons, one-way analysis of variance (ANOVA) or Kruskal-Wallis test was performed, followed by post hoc tests for pairwise comparisons. A significance level (α) of 0.05 was considered for all statistical tests. Statistical significance was denoted by *p < 0.05, **p < 0.01, and ***p < 0.001. Results are expressed as mean ± Standard Error of the Mean (SEM).

## Results

### Construction of post-anal incision pain rat model

**Fig. 1:**
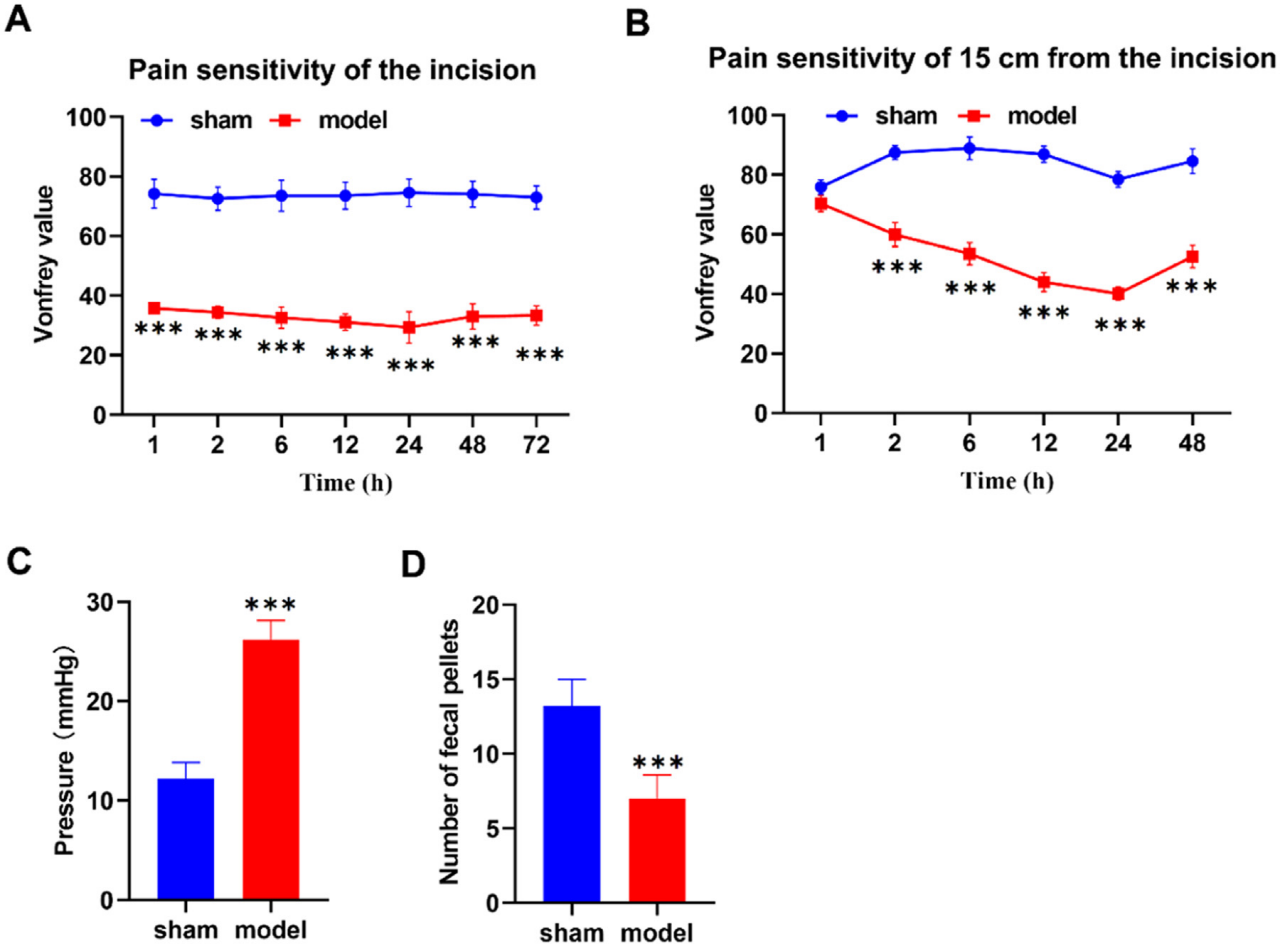
Construction of post-anal incision pain rat model. (A) Assessment of Mechanical Pain Sensitivity (Vonfrey) in the Sham and Model Groups. (B) Mechanical Pain Sensitivity (Vonfrey) at incision or a 15 cm Distant Area from the Incision. (C) Anal Sphincter Resting Pressure Measurements. (D) Number of Fecal Pellets Counted Postoperatively. *p < 0.05, **p < 0.01, ***p < 0.001 vs. sham group.

In the pursuit of elucidating the intricate mechanisms driving the effectiveness of electroacupuncture at the Chengshan acupoint for alleviating post-hemorrhoidectomy anal pain, a meticulously devised rat model mimicking post-anal incision pain was established, employing Sprague-Dawley (SD) rats as subjects. Pain sensitivity of the incision site (Vonfrey value against Time [h]) was depicted for both the sham and model groups, revealing a significantly higher sensitivity in the model group compared to the sham group (Fig. 1A). Similarly, the pain sensitivity 15 cm from the incision site (Vonfrey value against Time [h]) increased in the model group than the sham group (Fig. 1B). Furthermore, pressure dynamics (mmHg) in the anal region for both the sham and model groups demonstrated lower value in the sham group compared to the model group (Fig. 1C). Complementing these assessments, the number of fecal pellets excreted by the rats within the initial 6 hours post-surgery was meticulously documented, offering insights into potential alterations in bowel motility consequent to the surgical procedure, with the sham group surpassing the model group in count (Fig. 1D). This well-established rat model lays the foundation for comprehensive assessments, encompassing pain sensitivity, anal sphincter pressure, and early postoperative bowel function.

### Elevated expression of mAChRs in the post-anal incision pain rat model

**Fig. 2:**
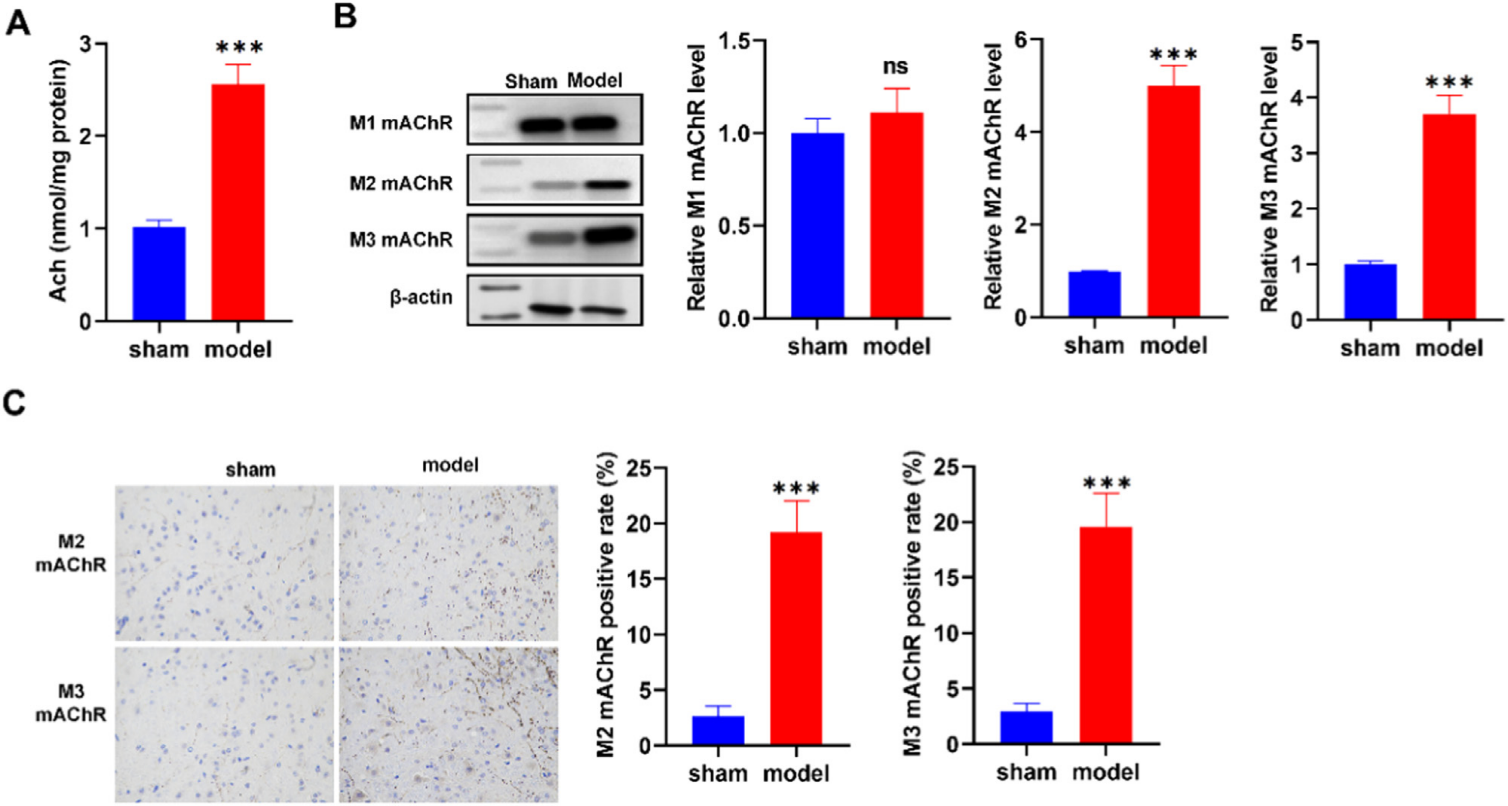
Enhanced mAChRs expression in post-anal incision pain rat model. (A) Elevated Acetylcholine (Ach) Levels in the Model Group. (B) Increased Expression of M2 mAChR Subtype. (C) Increased Expression of M3 mAChR Subtype. *p < 0.05, **p < 0.01, ***p < 0.001 vs. sham group.

This elevation aligns with potential pain-associated physiological responses. Employing western blot and immunohistochemistry techniques, it unveiled the expression patterns of distinct mAChR subtypes (M1, M2, and M3) (Fig. 2B and C). In both instances, heightened expression in M2, and M3 mAChR is evident in the model group relative to the sham group, illustrating a robust upregulation of mAChRs. This significant increase in mAChR expression, particularly in the M2 and M3 subtypes within the post-anal incision pain rat model, highlighted the potential involvement of cholinergic signaling pathways in pain sensitivity modulation.

### Significantly reduced anal pain intensity in mAChRs knockout mice

**Fig. 3:**
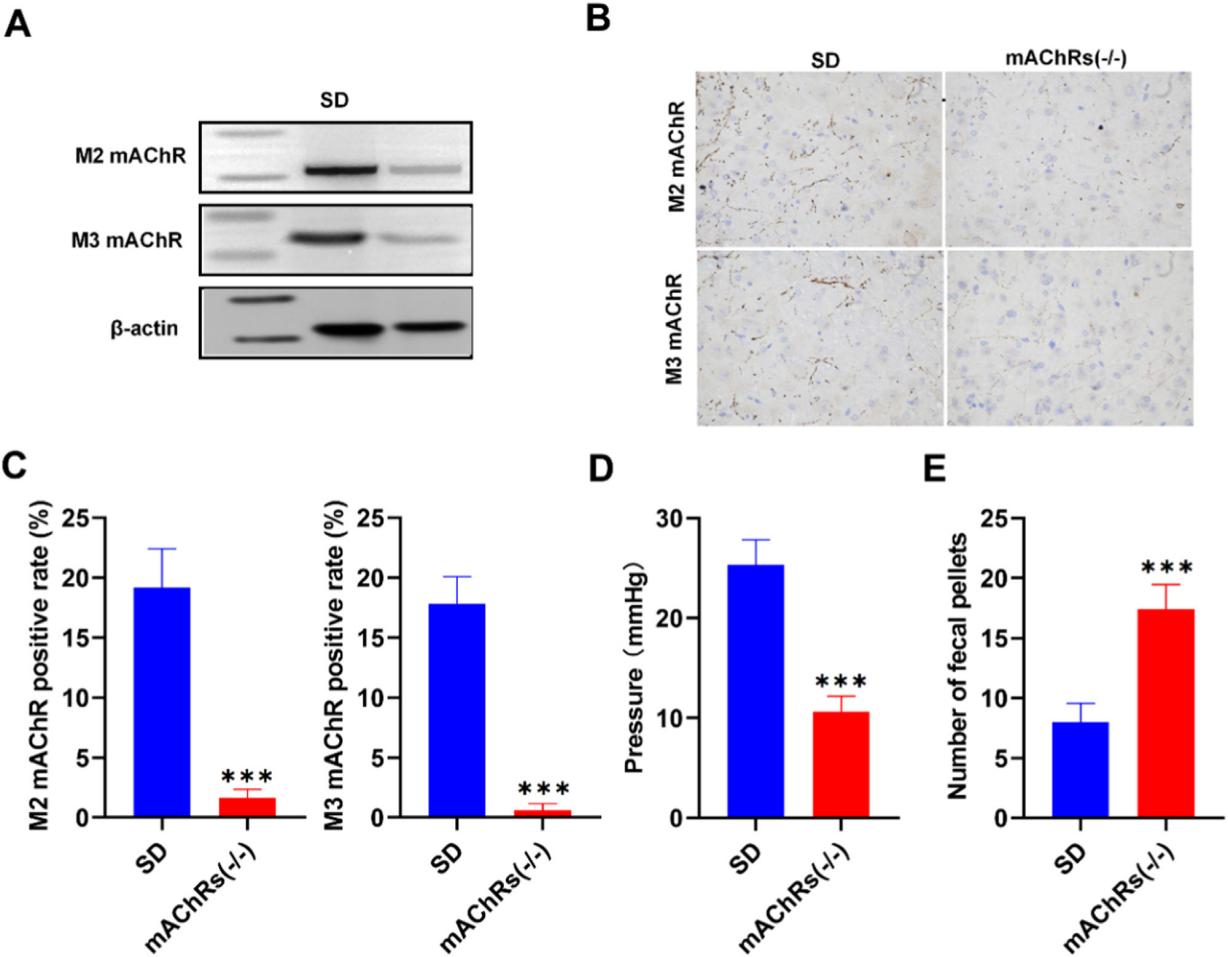
mAChRs knockout reduces anal pain sensitivity. (A) Diminished Pain Sensitivity in mAChRs Knockout Mice (SD group vs. mAChRs(-/-) group). (B) Western blot for M2 and M3 mAChR Expression. (C) Immunohistochemistry (IHC) for M2 and M3 mAChR Expression. (D) Positive Rate of M2 and M3 mAChRs. (E) Anal Sphincter Resting Pressure Measurements. (F) Number of Fecal Pellets Counted Postoperatively. *p < 0.05, **p < 0.01, ***p < 0.001 vs. SD group.

A model involving both mAChRs knockout and normal rats experiencing post-anal incision pain was established (SD group). Directly comparing the SD group with the mAChRs(-/-) group, Western Blot (WB) was taken to assess the expression of M2 and M3 mAChRs, revealing higher expression levels in the SD group compared to the mAChRs(-/-) group (Fig. 3A). Similarly, Immunohistochemistry (IHC) of samples extracted from striatal tissues confirmed this pattern of elevated M2 and M3 mAChR expression in the SD group relative to the mAChRs(-/-) group (Fig. 3B). Furthermore, the positive rates of M2 and M3 mAChRs were notably higher in the SD group compared to the mAChRs(-/-) group (Fig. 3C), supporting the notion of augmented cholinergic signaling in the SD group. Consistent with the above findings, anal pressure readings demonstrated higher levels in the SD group compared to the mAChRs(-/-) group (Fig. 3D), emphasizing the potential role of mAChRs in modulating anal sphincter dynamics. Moreover, the number of fecal pellets excreted by the rats showed a notable increase in the mAChRs(-/-) group compared to the SD group (Fig. 3E), suggesting that mAChRs play a role in influencing postoperative bowel motility. Collectively, these findings underscore the pivotal contribution of mAChRs to anal pain modulation.

### Regulation of IP3-Ca^2+^-CaM signaling pathway by mAChRs

**Fig. 4:**
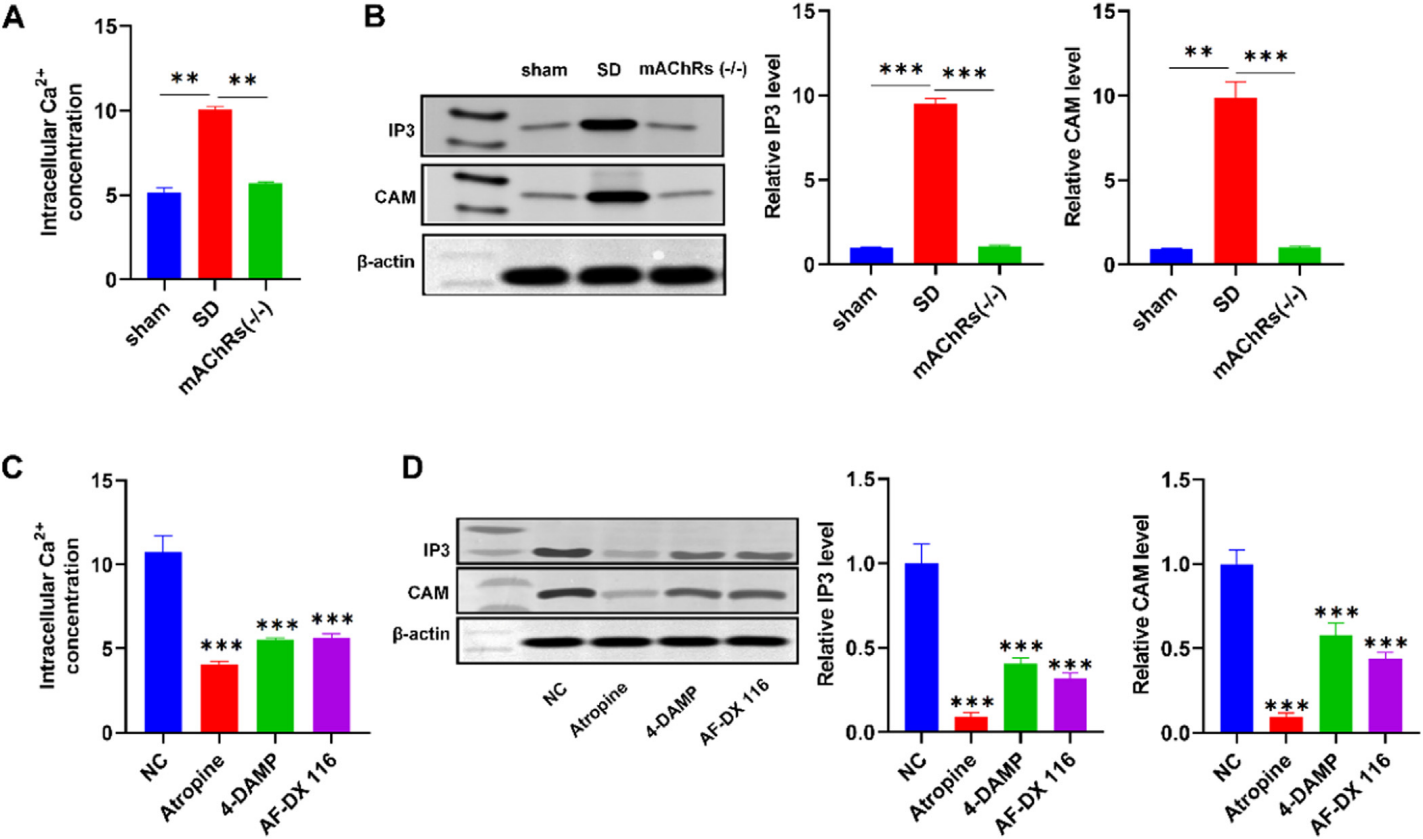
Regulation of IP3-Ca^2+^-CaM signaling by mAChRs and electroacupuncture. (A) Altered Intracellular Ca^2+^ Concentration in Different Groups. (B) Western blot for IP3 and CaM Expression. (C) Effects of mAChRs Antagonists on Intracellular Ca^2+^ Concentration. *p < 0.05, **p < 0.01, ***p < 0.001 vs. NC group.

The intracellular Ca^2+^ concentration is assessed, revealing lower levels in the sham group compared to the SD group, and higher levels in the SD group relative to the mAChRs(-/-) group (Fig. 4A). Western blot of IP3 (Inositol trisphosphate) and CaM (Calmodulin) expressions demonstrated lower levels in the sham group compared to the SD group, and higher levels in the SD group compared to the mAChRs(-/-) group (Fig. 4B). Moreover, rat anal sphincter smooth muscle cells were isolated, cultured, and subjected to various interventions with mAChRs antagonists: atropine, a broad mAChR antagonist; 4-DAMP, an M3 mAChR antagonist; and AF-DX 116, an M2 mAChR antagonist. Comparing the NC group with the Atropine, 4-DAMP, and AF-DX 116 groups, intracellular Ca^2+^ concentration demonstrated higher levels in the NC group relative to the mAChR antagonist groups (Fig. 4C). Similarly, IP3 and CaM expression detected by western blot revealed higher expression levels in the NC group compared to the mAChR antagonist groups (Fig. 4D). These findings collectively emphasized the regulatory role of mAChRs in modulating the IP3-Ca^2+^-CaM signaling cascade, providing insights into the intricate cellular mechanisms underlying the observed anal pain modulation.

### Alleviation by electroacupuncture treatment on postoperative anal pain in rat model

**Fig. 5:**
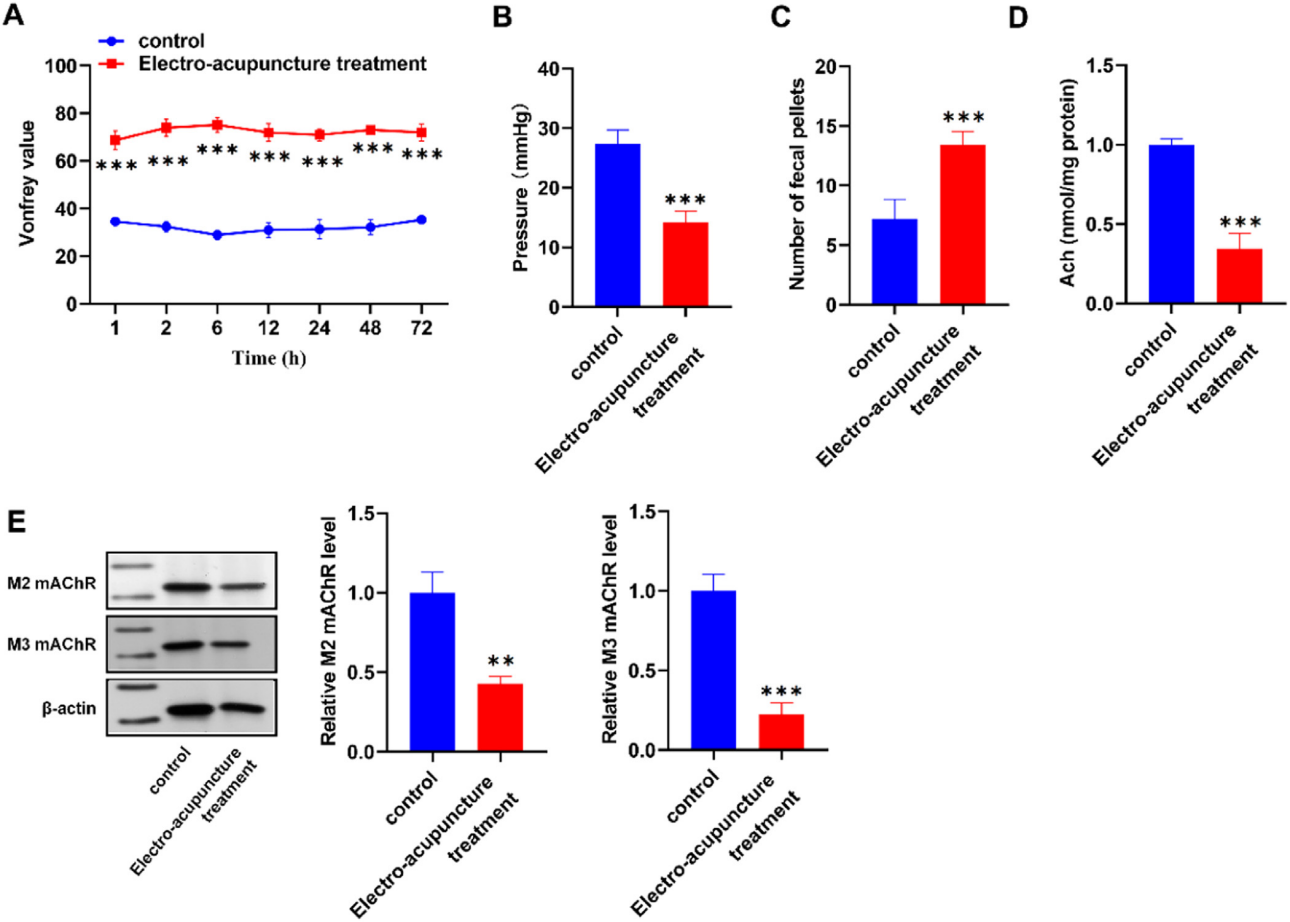
Electroacupuncture alleviates postoperative anal pain. (A) Reduced Pain Sensitivity (Vonfrey) following Electroacupuncture Treatment. (B) Altered Anal Sphincter Resting Pressure Measurements. (C) Improved Bowel Motility as Indicated by Number of Fecal Pellets. (D) Lowered Acetylcholine (Ach) Levels. (E) Western blot for M2 and M3 mAChR Expression. *p < 0.05, **p < 0.01, ***p < 0.001 vs. control group.

Comparing the control group with the electroacupuncture treatment group, the study involved the establishment of a post-anal incision pain model in normal Sprague-Dawley (SD) rats, followed by electroacupuncture intervention at the Chengshan acupoint. Pain sensitivity, as measured by Vonfrey value against Time (h), showcased significant alleviation (higher Vonfrey value) in the electroacupuncture treatment group compared to the control group (Fig. 5A). Anal pressure measurements displayed higher values in the control group relative to the electroacupuncture treatment group (Fig. 5B), indicating that electroacupuncture has a potential effect on anal sphincter relaxation. Furthermore, the number of fecal pellets excreted by the rats was notably increased in the electroacupuncture treatment group compared to the control group (Fig. 5C), indicating potential improvements in postoperative bowel motility. Acetylcholine (Ach) levels were significantly lower in the electroacupuncture treatment group compared to the control group (Fig. 5D), suggesting that electroacupuncture might influence cholinergic signaling. Additionally, Western blot for M2 and M3 mAChR expression revealed lower expression levels in the electroacupuncture treatment group compared to the control group (Fig. 5E), hinting at possible regulatory effects of electroacupuncture on mAChRs. These findings collectively indicated that electroacupuncture treatment at the Chengshan acupoint holds promise in alleviating postoperative anal pain.

## Discussion

The present study aimed to elucidate the mechanisms underlying the analgesic effects of electroacupuncture at the Chengshan acupoint in post-hemorrhoidectomy anal pain. The present findings shed light on the intricate interplay between cholinergic signaling, mAChRs expression, and pain modulation.

Electroacupuncture treatment emerged as a promising therapeutic strategy in the treatment of chronic pain disorders.^14^^,^^15^ A randomized controlled trial found that strong electroacupuncture was more effective in strengthening the Conditioned Pain Modulation (CPM) function than weak or sham electroacupuncture in knee osteoarthritis.^16^ In the present study's result, the observed improvements in pain sensitivity, anal pressure dynamics, fecal pellet count, and mAChR expression in response to electroacupuncture underscore its potential analgesic effects. This aligns with prior studies highlighting the role of electroacupuncture in pain relief.^17^

Central to the present investigation is the Chengshan acupoint, strategically located on the gastrocnemius muscle. Electroacupuncture at this acupoint offers a prospective solution for postoperative anal pain management. Studies have evaluated the effect of EA at Chengshan (BL 57), Dachangshu (BL 25), and Erbai (EX-UE 2) on postoperative complications of mixed hemorrhoids,^18^^,^^19^ as well as the clinical efficacy of EA at Changqiang (GV 1) and Chengshan (BL 57) on postoperative pain and discomforts in patients with mixed hemorrhoids.^20^ Despite the growing interest in its analgesic potential, the intricate mechanisms underpinning its effects remain an area of scholarly inquiry.

Another research put forward that specifically in hemorrhoidectomy, different frequencies of electroacupuncture preconditioning at Xialiao and Chengshan act diversely on anal pain after Milligan-Morgan hemorrhoidectomy.^21^ However, it did not manage to explore the molecular mechanism within the whole relief reaction. In the present results, as the discovery of downregulation of M2 and M3 mAChRs following electroacupuncture, an unprecedented link between the IP3-Ca^2+^-CaM signaling pathway and the analgesic effects of electroacupuncture was established. mAChRs emerged as key players in pain modulation. The significant upregulation of mAChRs, particularly the M2 and M3 subtypes, within the post-anal incision pain rat model, points toward their potential involvement in pain sensitivity. This observation aligns with previous research linking mAChRs to pain pathways.^22^, ^23^, ^24^ For example, a study published in Frontiers in Cardiovascular Medicine found that chronic pain stress elevated the protein expression of muscarinic acetylcholine receptor M2 in the atria of mice.^25^ Another study published in SpringerLink discussed the behavioral and molecular basis of cholinergic modulation of pain, with a focus on nicotinic acetylcholine receptors.^26^ The authors found a similar outcome that mAChRs knockout in rats significantly reduced post-anal incision pain. Analyses of M2 and M3 mAChR expression revealed their prominent role in pain modulation. The present investigation further delved into the regulatory effects of mAChRs on the IP3-Ca^2+^-CaM signaling pathway. The alterations in intracellular calcium concentration, IP3, and CaM expression underscore the potential role of mAChRs in influencing cholinergic signaling cascades. These findings resonated with another study demonstrating the M3-AChR-mediated IP3/Ca^2+^/PKC pathway.^27^

While the present study provides valuable insights, several limitations warrant consideration. The rat model, though well-established, may not fully capture human complexities. The exclusivity of the M2 and M3 mAChR subtypes studied may overlook the potential involvement of other subtypes. Electroacupuncture's exact influence on IP3-Ca^2+^-CaM pathway dynamics necessitates further exploration.

In conclusion, this study offers novel insights into the intricate mechanisms by which electroacupuncture at the Chengshan acupoint alleviates postoperative anal pain. The multifaceted interplay between cholinergic signaling, mAChRs expression, and downstream signaling pathways provides a foundation for future investigations in pain management strategies.

## Availability of data and materials

The datasets used and analyzed in the current study will be available from the corresponding author upon request.

## Authors’ contributions

Tianshu Xu and Yujuan Wang designed the study and provided theoretical guidance, Yang Song wrote and revised the manuscript. Yang Wang collected data. Ming Li analyzed data. Tianshu Xu and Yujuan Wang contributed equally to this work and are co-corresponding authors. All authors read and approved the final submitted manuscript.

## Declaration of competing interest

The authors declare no conflicts of interest.
