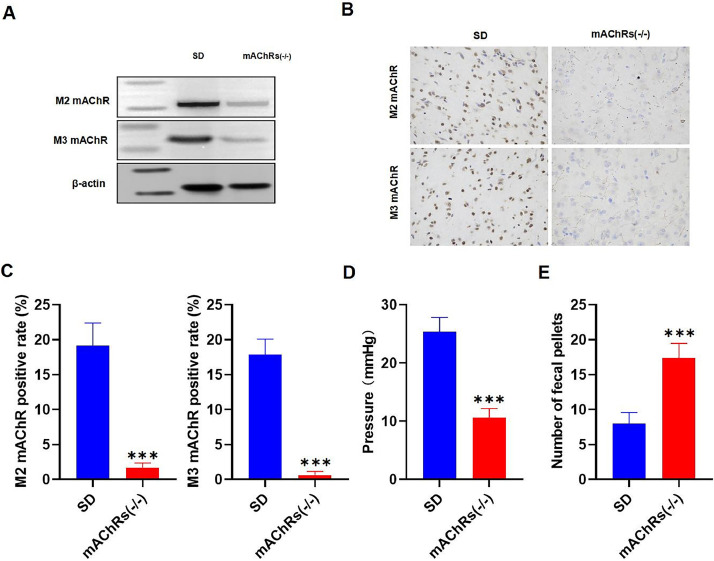# Corrigendum to “Exploration of the mechanism underlying the therapeutic effect of electroacupuncture at chengshan acupoint on post-hemorrhoidectomy anal pain: Insights from the mAChRs/IP3-Ca2+-CaM signaling pathway” [CLINSP 79 (2024) 10048]

**DOI:** 10.1016/j.clinsp.2024.100568

**Published:** 2025-01-07

**Authors:** Yang Song, Yang Wang, Ming Li, Yujuan Wang, Tianshu Xu

**Affiliations:** a Department of Traditional Chinese Medicine, Nanjing Drum Tower Hospital Clinical College of Nanjing University of Chinese Medicine, Nanjing, Jiangsu, China; b Department of Traditional Chinese Medicine, Nanjing Drum Tower Hospital, Nanjing, Jiangsu, China

The authors regret that the above article contained an error in Fig. 3B. The authors would like to apologize for any inconvenience caused. Please note that the error does not affect the key findings and conclusions of the article, and all authors favour publishing this *Corrigendum*.

*Corrigendum* to Fig. 3B: Fig. 3B has been corrected as follows:

**Figure:**